# Osmotic perception of GABAergic synaptic transmission in the supraoptic nucleus of rats

**DOI:** 10.1016/j.ibror.2020.06.007

**Published:** 2020-07-02

**Authors:** Toyoaki Ohbuchi, Takeshi Saito, Toru Yokoyama, Hirofumi Hashimoto, Takashi Maruyama, Hideaki Suzuki, Yoichi Ueta

**Affiliations:** a Department of Physiology, School of Medicine, University of Occupational and Environmental Health, Kitakyushu, Japan; b Department of Otorhinolaryngology-Head and Neck Surgery, School of Medicine, University of Occupational and Environmental Health, Kitakyushu, Japan; c Department of Neurosurgery, School of Medicine, University of Occupational and Environmental Health, Kitakyushu, Japan

**Keywords:** Hypertonic condition, Hypotonic stimulation, Miniature GABAergic postsynaptic currents (mGPSCs), Supraoptic nucleus, Vasopressin neuron, Whole-cell patch-clamp

## Abstract

Extracellular osmolality plays a crucial role in controlling the activation of neurons. Hypertonic stimulation modulates glutamatergic inputs to the supraoptic nucleus (SON) magnocellular neurosecretory cells (MNCs) putative vasopressin (VP) neurons through capsaicin-insensitive transient receptor potential vanilloid (TRPV) 1 channels on the presynaptic terminals. However, it remains unclear whether osmotic stimulation modulates GABAergic inputs to VP-secreting neurons within punched-out slices containing only the SON and the perinuclear zone.

To answer this question, we studied the effects of various osmotic conditions on the miniature GABAergic postsynaptic currents (mGPSCs) using the whole-cell patch-clamp technique on rat SON putative VP-secreting neurons in small slice preparations.

We revealed that incubation in hypertonic solution for 2 h reduced both the frequency and amplitude of the mGPSCs to the SON putative VP neurons, whereas the mGPSCs were unaffected when the external osmolality was changed from isotonic to hypotonic. Of interest, we found that changing from a hypertonic to hypotonic environment increased the frequency of the mGPSCs. This effect was independent of TRPV4.

We hypothesize that two coordinated mechanisms may play an important role in the regulation of a wide range of physiological functions of VP.: 1) the modulation of GABA_A_ receptor properties by brain-derived neurotrophic factor (BDNF)-induced tyrosine kinase B receptor-mediated signaling under hypertonic conditions, and 2) cell swelling-induced activation of whole-cell anion currents under hypotonic conditions.

## 1 Introduction

Magnocellular neurosecretory cells (MNCs) in the supraoptic nucleus (SON) synthesize and secrete arginine vasopressin (VP) and oxytocin (OT). It has long been known that VP which is released into the systemic circulation contributes a regulatory mechanism of body fluid homeostasis. Recently, the possibility that VP may be somatodendritically released into the brain, and may play an important role in social behavior, sexual motivation and pair bonding, and maternal responses to stress (Insel, 2010). The release of VP is closely related to the electrical activity of MNCs, which is modulated by neurotransmitters and neuromodulators. Fast synaptic inputs (from glutamate and GABA_A_ receptors, respectively) are two of the most important regulators of MNC electrical activity (Leng et al., 1999; Shibuya et al., 2000). In general, neuronal excitability in the adult brain is controlled by a balance between synaptic excitation and inhibition mediated by glutamate and GABA, respectively. However, a recent report revealed that GABA is excitatory in adult VP neurons under normal condition (Haam et al., 2012).

Previous studies have suggested that transient receptor potential vanilloid (TRPV) cation channels sense the extracellular osmotic environment such as hypertonicity and hypotonicity (Liedtke and Friedman, 2003; Mizuno et al., 2003; Alessandri-Haber et al., 2005; Liedtke, 2007). Our previous electrophysiological study found that short term hypertonic stimulation directly enhances glutamatergic inputs to the SON MNCs’ putative VP-secreting neurons (hereinafter referred to as VP neurons) through capsaicin-insensitive TRPV1 channels on the presynaptic terminals (Yokoyama et al., 2010). However, it remains unclear whether osmotic stimulation affects miniature GABAergic postsynaptic currents (mGPSCs) to the SON VP neurons through osmosensors such as TRPV on the presynaptic terminal and/or on the postsynaptic cell membrane, which lead increase of intracellular Ca^2+^ (Balena et al., 2010).

In this work, we investigated the effects of various osmotic conditions on mGPSCs using a whole-cell patch-clamp technique on VP neurons in rat SON slice preparations. We revealed that incubation in hypertonic solution for 2 h reduced both the frequency and amplitude of the mGPSCs to the SON VP neurons. Further, we found that the frequency of the mGPSCs increased after an abrupt drop from hyper- to hypo- extracellular osmolality and that this increase was not dependent on TRPV4. The mGPSCs were unaffected when the external osmolality was changed from isotonic to hypotonic. Although the specific biologic mechanisms of this effect remain unclear, prior evidence has suggested that hypertonic-induced brain-derived neurotrophic factor (BDNF) release and hypo-osmotic cell swelling-induced activation of whole-cell anion currents may be involved in this process.

## 2 Experimental procedures

### 2.1 Incubation solution

The cutting solution during preparation was a modified Krebs-Henseleit solution (KHS) containing (mM): NaCl, 124; KCl, 5; MgSO_4_, 1.3; KH_2_PO_4_, 1.24; CaCl_2_, 2; NaHCO_3_, 25.9; and glucose, 10. The solution used in experiments was another modified KHS containing (mM): NaCl, 100; KCl, 5; MgSO_4_, 1.3; KH_2_PO_4_, 1.24; CaCl_2_, 2; NaHCO_3_, 25.9; and glucose, 10. Osmolalities for the hypotonic, isotonic, and hypertonic conditions were adjusted to 250 ± 3, 275 ± 3, 300 ± 3, and 350 ± 3 mOsmol/kg with mannitol, respectively. The solution was continuously oxygenated with a mixture of 95 % O_2_ and 5% CO_2_. The pipette solution used in the recording electrodes contained (mM): K-gluconate, 140; MgCl_2_, 1; CaCl_2_, 1; EGTA, 10; HEPES, 10; Mg-ATP, 2; pH 7.3 with Tris base.

### 2.2 Sampling of supraoptic nucleus slice

All tissue preparations and electrophysiological experiments were performed as previously reported (Ohbuchi et al., 2009; Yokoyama et al., 2010). Experiments were performed on young adult (3∼4-week-old) male Wistar rats weighing 80−150 g. The animals were housed in standard plastic cages at 23–25 °C in a 12-h light/dark cycle. All experiments in this study were carried out in accordance with the Physiological Society of Japan under the guidance of the Ethics Committee of Animal Care and Experimentation, University of Occupational and Environmental Health, Japan.

The rats were sacrificed by decapitation, and care was taken to avoid gross contusion or hemorrhage during and after removal of the brain. Brains were quickly removed and cooled in a perfusion medium at 4 °C for 1 min. A block containing the hypothalamus was cut from the brain and glued onto the stage of a vibratome-type slicer (Linearslicer Pro 7, DSK, Kyoto, Japan). After the meninges were carefully removed, coronal slices (150 μm) containing SON were cut from the block in the medium at 4 °C. The slices were carefully trimmed with a circular punch (inner diameter 1.8 mm) and incubated in the isotonic (300 mOsmol/kg) or hypertonic (350 mOsmol/kg) medium at room temperature (22–24 °C) for 2 h until they were used for electrophysiological measurements.

### 2.3 *In vitro* slice patch-clamp and data analysis

The slice was placed on a glass-bottom chamber and fixed with a grid of parallel nylon threads supported by a U-shaped stainless-steel weight. The volume of the recording chamber was 1 ml and the perfusion rate was 2 ml/minute. Continuous perfusion was performed using an eight-head peristaltic pump (MP-8, Gilson nucleus, Villiers le Bel, France). The solution volume was kept constant by a low-pressure aspiration system. Slices were moved from an incubation solution to chamber filled with the same osmotic solution. The perfusion solutions contained 1 μM TTX. To identify magnocellular neurons in the SON, we used an upright microscope (Axioskop, ZEISS, Germany) with Nomarski optics (×400). All solutions were applied to the slice preparations by using a two-way valve to switch the perfusion solution from the control buffer to a buffer with various osmolality and 4-Phorbol 12, 13-dicaprinate (4α-PDD) (HV 4-4, Hamilton, Reno, NV, USA). The electrodes used in this study were triple-pulled (P-87, Sutter Instrument Co., Novoto, CA, USA) from a glass capillary. The pipettes had a final resistance of 5–8 MΩ when filled. Whole-cell recordings were made from microscopically identified SON neurons in the upper surface layers of the slices at room temperature (22–24 °C). A previous immunohistochemical study demonstrated that VP neurons are more common in the caudal and ventral parts of the SON (Rhodes et al., 1981). Moreover, according to the VP-enhanced green fluorescent protein (VP-eGFP) fusion gene/OT-monomeric red fluorescent protein 1 (OT-mRFP1) fusion gene double transgenic rat, the fluorescence of eGFP was observed in the ventral side, whereas the fluorescence of mRFP1 was observed in the dorsal side of the SON (Kato et al., 2011). Therefore, we recorded mGPSCs in the ventral part of the SON MNCs. Currents were recorded with an EPC-9 amplifier (HEKA, Lambrecht, Germany). Signals were digitized with an analog-digital converter. (MacLab/v. 3.5, Castle Hill, Australia) (Ohbuchi et al., 2009) . Only the AC components (selected using a 1-Hz high pass filter) were used for quantitative analyses of synaptic currents in AxoGraph v.3.6.1 (Axon Instruments, Foster Hill, CA, USA). Spontaneous events were automatically screened using an amplitude threshold of 15 pA and were then visually accepted or rejected based on the rise and decay time. Recordings of postsynaptic currents were begun over 5 min after membrane rupture at a holding potential of −20 mV when the currents reached a steady state. Recordings were collected during periods of stable series resistance. The average values of the frequency and amplitude of mGPSCs during the initial 10 min were calculated as baseline values. Following the initial 10 min, the stimulating solution was perfused for 10 min. And then, the wash-out solution which is the same solution as the initial solution was perfused for 10 min. The average frequency and amplitude values for the first 10 min after adding the new buffer were normalized to the baseline values. All data were expressed as mean ± standard error of the mean (SEM). Results were compared by Student’s *t*-test or Welch’s *t*-test. P<0.05 was considered statistically significant. The number of neurons tested is represented as ‘n’.

## 3 Results

### 3.1 Hypertonic conditions reduce the frequency and amplitude of mGPSCs

In our previous experiment, we have revealed that short-term (5 min) hypertonic stimulation affected neither the frequency nor the amplitude of the mGPSCs in rat SON slice preparation which is the same-size sample we used in the present study (Yokoyama et al., 2010).

**Fig. 1:**
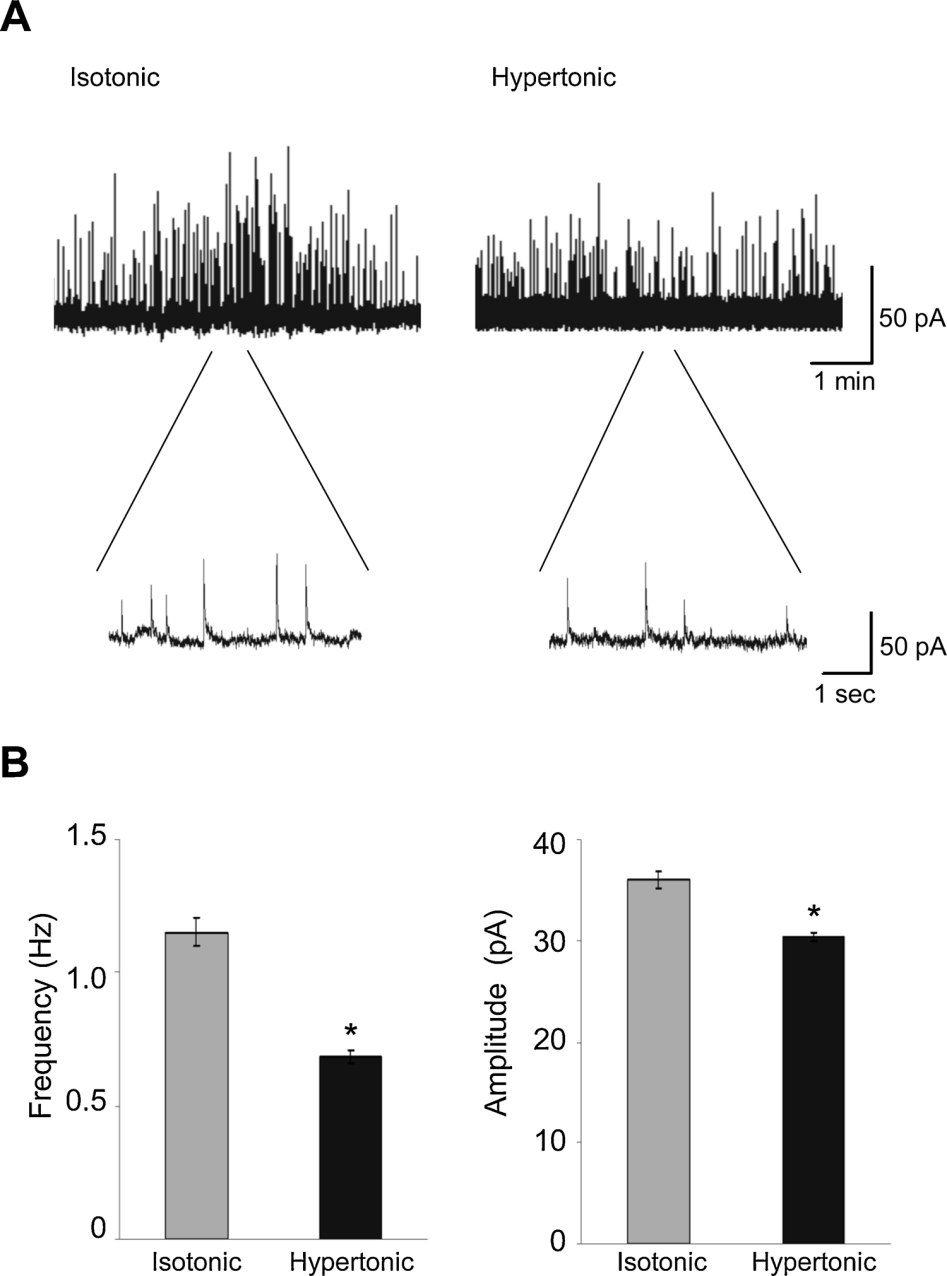
(A, representative examples of the miniature GABAergic postsynaptic currents (mGPSCs) after 2 h incubation in the isotonic (300 mOsmol/kg) and hypertonic (350 mOsmol/kg) condition are shown. (B, under persistent hypertonic conditions, both the frequency (isotonic: 1.15 ± 0.05 Hz, n = 19; hypertonic: 0.68 ± 0.03 Hz, n = 32, p < 0.05) and the amplitude (isotonic: 36.1 ± 0.78 pA, n = 19; hypertonic: 30.4 ± 0.38 pA, n = 32, p < 0.05) of mGPSCs were significantly smaller relative to isotonic conditions.

To assess whether longer-term (2 h) incubation in a hypertonic solution (350 mOsmol/kg) alters mGPSCs, we measured the frequency and amplitude for cells incubated in an isotonic (300 mOsmol/kg) or the hypertonic solutions. Interestingly, in the hypertonic condition, both the frequency (isotonic: 1.15 ± 0.05 Hz, n = 19, hypertonic: 0.68 ± 0.03 Hz, n = 32, p < 0.05) and the amplitude (isotonic: 36.1 ± 0.78 pA, n = 19, hypertonic: 30.4 ± 0.38 pA, n = 32, p < 0.05) of mGPSCs were significantly smaller (Fig. 1).

### 3.2 Effects of decreasing extracellular osmolality on mGPSCs in SON MNCs

**Fig. 2:**
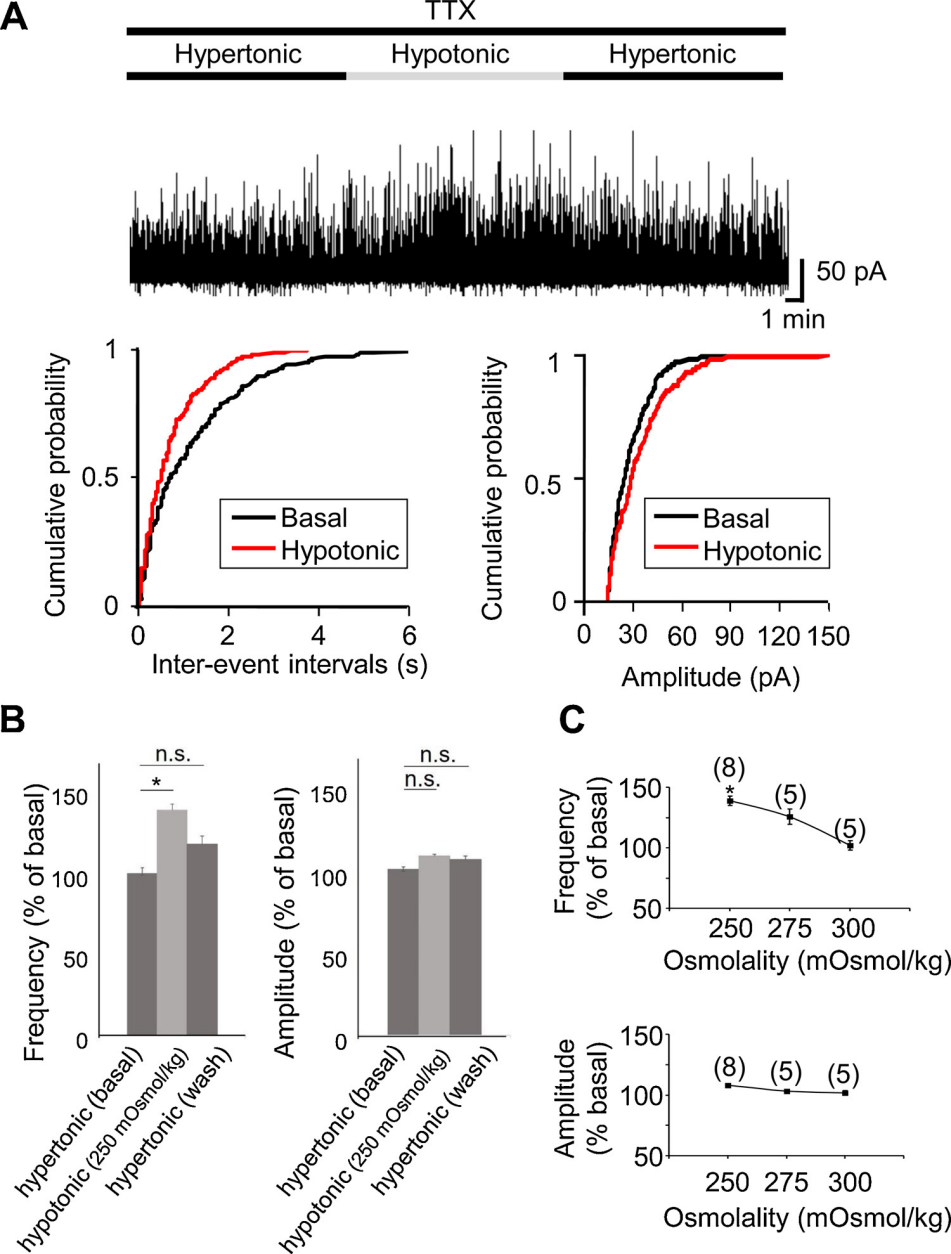
From the hyper- to hypotonic stimulation increased the frequency of the mGPSCs. (A, representative example, cumulative probability for inter-event interval and amplitude in the case of 250 mOsmol/kg are shown. (B, the average time-courses of the frequency and amplitude in mGPSCs are shown. (C, the increase in frequency due to hypotonic stimulation showed an osmolality-dependent relationship: 250 mOsmol/kg (139 ± 3.9 % of baseline, p < 0.05, n = 8), 275 mOsmol/kg (126 ± 6.4 % of baseline n = 5, p > 0.05), and 300 mOsmol/kg (102 ± 3.9 % of baseline, n = 5, p > 0.05), whereas the amplitude was not significantly altered (108 ± 1.2 % of baseline for 250 mOsmol/kg, n = 8, p > 0.05: 103 ± 1.7 % of baseline for 275 mOsmol/kg, n = 5, p > 0.05: 102 ± 1.8 % of baseline for 300 mOsmol/kg, n = 5, p > 0.05).

To determine the effects of an abrupt drop from hyper to hypo extracellular osmolality on GABAergic postsynaptic inputs, cells were first incubated for 2 h with hypertonic solution and then rapidly switched into either hypotonic (250 mOsmol/kg or 275 mOsmol/kg) or isotonic conditions. We measured the frequency and the amplitude of mGPSCs at the 10 min baseline, during the 10 min of the second incubation, and then followed by the 10 min wash-out with hypertonic solution. Hypotonic stimulation rapidly increased the frequency of the mGPSCs in an osmolality-dependent manner, where lower osmolality was associated with a larger increase in mGPSC frequency relative to baseline (Fig. 2). Indeed, we measured frequencies at osmolalities of 250 mOsmol/kg (139 ± 3.9 % of baseline, n = 8, p < 0.05), 275 mOsmol/kg (126 ± 6.4 % of baseline, n = 5, p > 0.05), and 300 mOsmol/kg (102 ± 3.9 % of baseline, n = 5, p > 0.05: Fig. 2C). However, the amplitude was not significantly altered (108 ± 1.2 % of baseline for 250 mOsmol/kg, n = 8, p > 0.05: 103 ± 1.7 % of baseline for 275 mOsmol/kg, n = 5, p > 0.05: 102 ± 1.8 % of baseline for 300 mOsmol/kg, n = 5, p > 0.05: Fig. 2C). In the case of 250 mOsmol/kg, the increased frequency by hypotonic stimulation rapidly decreased back near the baseline during wash-out (frequency; 117 ± 3.9 % of baseline, n = 8, p > 0.05: amplitude; 107 ± 2.1 % of baseline, n = 8, p > 0.05: Fig. 2B).

**Fig. 3:**
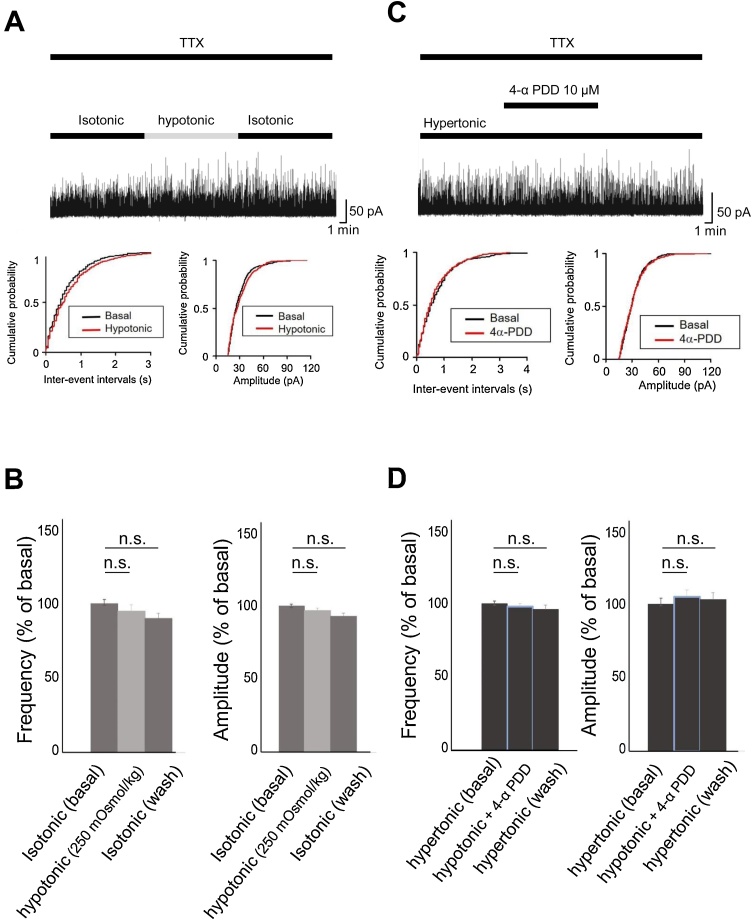
Hypotonic (250 mOsmol/kg) stimulation for 10 min changed after isotonic (300 mOsmol/kg) solution led to no changes in the frequency (94.9 ± 4.3 % of baseline, n = 5, p > 0.05) or amplitude (96.5 ± 1.9 % of baseline, n = 5, p > 0.05) of mGPSCs. Representative example, cumulative probability for inter-event interval and amplitude, and average time-course of the frequency and amplitude are shown in A and B, respectively. Neither the frequency nor the amplitude of the mGPSCs were affected after treatment with 10 μM 4α-PDD, a TRPV4 agonist, under hypertonic conditions (frequency; 105 ± 4.8 % of baseline, n = 4, p > 0.05: amplitude; 98.0 ± 2.2 % of baseline, n = 4, p > 0.05). Representative example, cumulative probability for inter-event interval and amplitude, and average time-course of the frequency and amplitude are shown in C and D, respectively.

To compare the effects of hyper- and isotonic incubation, mGPSCs were kept in isotonic conditions for 2 h and then abruptly switched to hypotonic conditions (250 mOsmol/kg) for 10 min. This change did not affect either the frequency (94.9 ± 4.3 % of baseline, n = 5, p > 0.05) or the amplitude (96.5 ± 1.9 % of baseline, n = 5, p > 0.05) of the mGPSCs (Fig. 3A and B).

### 3.3 TRPV4 activation alone does not increase mGPSC frequency

TRPV4 is typically activated by the swelling that occurs when cells are exposed to a hypotonic environment, resulting in increased intracellular Ca^2+^ (Liedtke et al., 2000). It can also be activated by a number of chemical compounds (Vriens et al., 2007). To test whether activation of TRPV4 results in changes in the mGPSCs, we treated cells with 10 μM 4α-PDD, a TRPV4 agonist, for 10 min in hypertonic conditions and measured changes in the frequency and the amplitude of mGPSCs. As of above, abruptly switching to hypotonic conditions did increase the frequency of the mGPSCs under hypertonic condition, but not under isotonic condition. Therefore, we investigated whether TRPV4 is involved the increase of frequency under hypertonic conditions. As a result, neither the frequency nor the amplitude of the mGPSCs were affected by treatment with 4α-PDD (frequency; 105 ± 4.8 % of baseline, n = 4, p > 0.05: amplitude; 98.0 ± 2.2 % of baseline, n = 4, p > 0.05: Fig. 3C and D).

## 4 Discussion

In this study, we revealed that incubation in hypertonic conditions for 2 h reduced both the frequency and amplitude of the mGPSCs. In addition, we found that the frequency of mGPSCs increased when extracellular fluids were changed from hypertonic to hypotonic, but the amplitude was not affected. These changes in mGPSCs frequency occurred in an osmolality-dependent manner, where greater reductions in osmolality were associated with greater increases in frequency. TRPV4 did not appear to contribute to this mechanism.

Previous research has shown that 15 min of hyperosmotic stress increases brain-derived neurotrophic factor (BDNF) mRNA and protein expression and leads to dendritic BDNF release in the SON of rats *in vivo* (Aliaga et al., 2002; Arancibia et al., 2007). BDNF activates postsynaptic tyrosine kinase (Trk) B receptors, decreasing the surface expression of GABA_A_ receptors and thus altering synaptic inhibition (Brunig et al., 2001; Jovanovic et al., 2004; Hewitt and Bains, 2006). Activation of the TrkB receptor and its downstream signaling pathway may trigger phosphorylation cascades that may be the mechanistic link between the attenuation of GABA responses and the internalization of GABA_A_ receptors (Cheng and Yeh, 2003).

In agreement with these findings, our previous research demonstrated a decrease in both the frequency and amplitude of GPSCs after BDNF treatment of SON slice preparations (Ohbuchi et al., 2009). In addition, postsynaptic responses to focal GABA application were significantly attenuated after BDNF treatment of dissociated cultures of SON MNCs expressing VP-enhanced green fluorescent protein (Ueta et al., 2005; Ohbuchi et al., 2009). Thus, it appears that hyperosmotic conditions lead to the local release of BDNF in the SON, eventually inhibiting GABAergic postsynaptic inputs. The activation of the TrkB receptor and its downstream phosphorylation cascade may lead to changes in affinity or channel conductance and the internalization of GABA_A_ receptors, attenuating the inhibitory response. In this work, we found that GABAergic postsynaptic inputs were attenuated after 2 h of exposure to hypertonic solution alongside increases in local BDNF expression and release.

Next, we revealed that hypotonic stimulation after hypertonic incubation increased mGPSCs frequency. Previous studies have shown that hypotonic conditions inhibit the firing of VP neurons and that GABA-mediated GPSCs play an essential role in this mechanism (Richard and Bourque, 1995; Wang et al., 2013). However, these studies were performed using brain explants containing the organum vasculosum of the lamina terminalis, glia, and hypothalamic SON. To our knowledge, this study is the first to report that hypotonic stimulation increases the frequency of mGPSCs in a rat SON punched-out small slice preparation.

Once phosphorylated, the TrkB receptor and its associated downstream signaling proteins remain activated for hours, even after removal of BDNF (Choi et al., 2001). Indeed, we previously elucidated that the effects of BDNF on GPSCs were still present 30 min after removal of BDNF (Ohbuchi et al., 2009). Therefore, it is not likely that hypotonic stimulation reversed the effects of BDNF on mGPSCs.

A previous study reported that exposure to a hypotonic solution simultaneously induces cell swelling and activates whole-cell anion currents in dissociated rat SON VP neurons (Sato et al., 2011). This cell swelling-induced activation of whole-cell anion currents was observed immediately after hypotonic treatment (Sato et al., 2011). Substance of the anion channels activated during hypotonic-induced cell swelling have not been identified for now, whereas it is known that aquaporin channels play important roles in H_2_O influx (Giuliani and Peri, 2014). In light of this finding, the inhibition of GABA_A_ receptors upon switching from a hyper- to hypotonic environment may be partially driven by increased susceptibility to Cl^−^ influx during cell swelling.

In the present study, mGPSCs to the SON MNCs were unaffected by changes both from iso- to hypotonic and from hyper- to isotonic external osmolality. These findings were consistent with the idea that there would be no increase in BDNF expression or cell swelling in the isotonic condition.

**Fig. 4:**
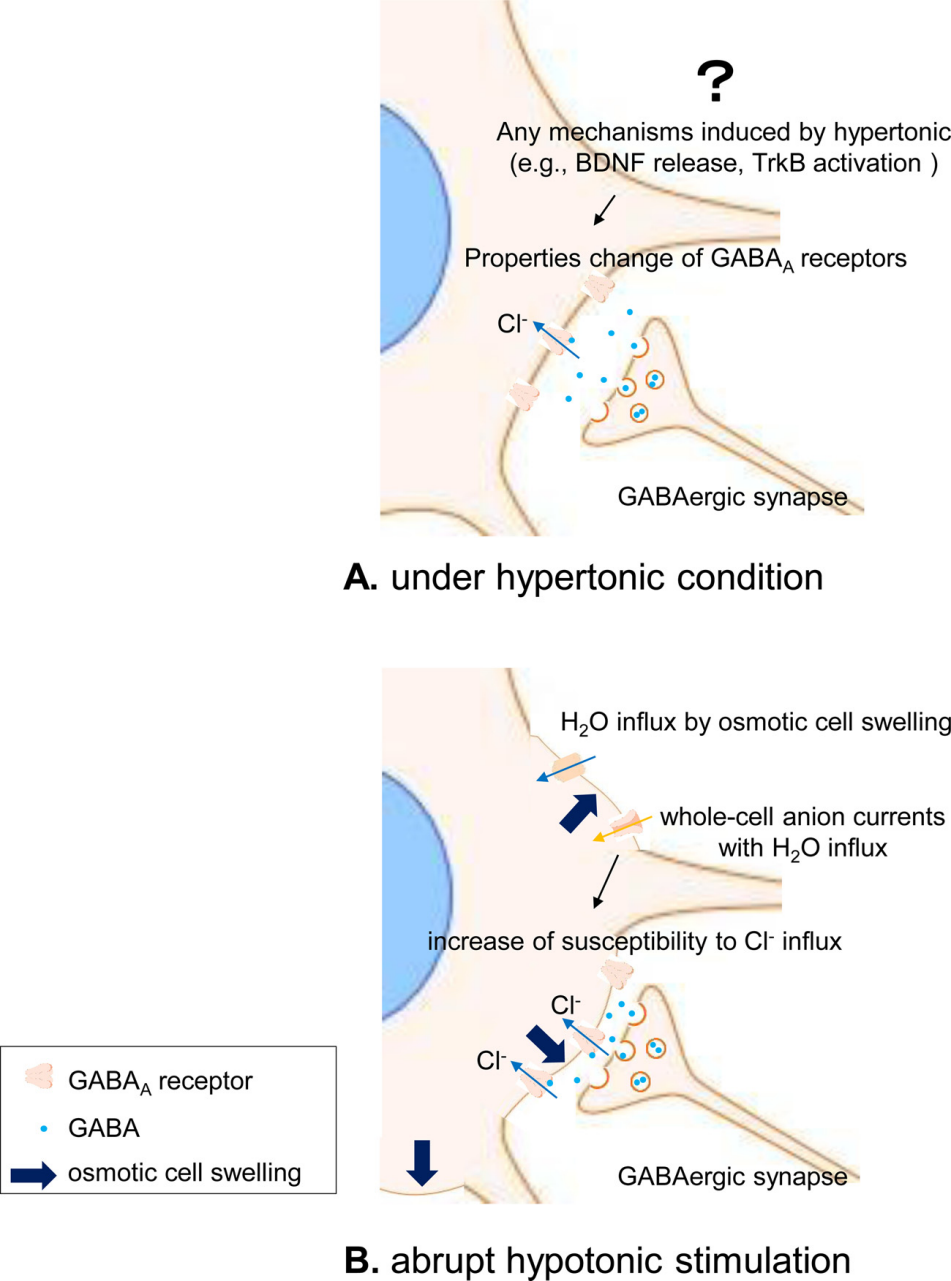
Schematic of our proposed mechanisms for the increase the GABAergic postsynaptic inputs by the hypotonic stimulation after hypertonic condition. (A, under hypertonic conditions, BDNF are expressed and release in local SON. BDNF-induced TrkB receptor-mediated signaling modulates GABA_A_ receptor properties under hypertonic conditions. (B, exposure to a hypotonic solution simultaneously induces cell swelling and activates whole-cell anion currents which may lead to increase a susceptibility of GABA_A_ receptors to Cl^−^ under hypotonic conditions.

In conclusion, we hypothesize that two coordinated mechanisms regulate homeostasis in response to changing osmolality: 1) BDNF-induced TrkB receptor-mediated signaling modulates GABA_A_ receptor properties under hypertonic conditions, and 2) cell swelling-induced activation of whole-cell anion currents may lead to increasing susceptibility of GABA_A_ receptors under hypotonic conditions (Fig. 4).

　In the present study, certain issues and limitations remain unresolved. First, the hypothesis we propose is preliminary and speculative, so that other various mechanisms cannot be ruled out. For example, it’s possible the involvement of glial cells in responses observed in the current experiments. Various stimulation including the hypertonicity-induced glutamate release may retract the astrocytes process which change the microenvironment (Ohbuchi et al., 2015). The hypothesis might be also limited statistically due to small size of the samples. Second, we cannot definitely affirm that MNCs recorded from the ventral parts of the SON are authentic VP-secreting neurons. Third, hypertonic conditions could affect its response to the TRPV4 agonist 4α-PDD. It was shown that the hypertonic condition can also affect the TRPV4 functions as a transducer of tonic stimulation (Alessandri-Haber et al., 2005; Liedtke and Friedman, 2003; Mizuno et al., 2003). Finally, we cannot be completely confident that these findings reflect the physiologic properties of GABA effects *in vivo*. In general, the GABA action is excitatory in immature neurons. This is because the intracellular Cl^−^ concentration ([Cl^−^]i) is high, owing to high levels of the Na^+^-K^+^-2Cl^−^ cotransporter (NKCC1), which mediates inward transport of Cl^−^, and to low levels of the K^+^-Cl^−^ cotransporter (KCC2), which excludes Cl^−^ from the cell. In most neurons, the GABA response switches from excitation to inhibition during early postnatal development, due to the developmental decrease of the NKCC1 and increase of the KCC2 (Watanabe et al., 2014). However, GABA is excitatory in adult VP neurons under normal condition, suggesting that the classical excitation/inhibition paradigm of synaptic glutamate and GABA control of neuronal excitability does not apply to VP neurons　(Haam et al., 2012). The unique characteristics of VP neurons make an understanding more complicated.

　Despite these limitations, we believe that our hypothesis is potentially plausible. Further studies of the exact mechanistic details of these interactions are required to improve our understanding of fluid homeostasis regulation in the SON.

## Conflicts of interest

The authors have no conflicts of interest to disclose

## Acknowledgements/Funding

This study was supported by Grant-in-Aid for Scientific Research (B), No 17H04027 to Y.U. from the Japan Society for the Promotion of Science (JSPS).
